# Interaction of Gold Nanorods with Human Dermal Fibroblasts: Cytotoxicity, Cellular Uptake, and Wound Healing

**DOI:** 10.3390/nano9081131

**Published:** 2019-08-06

**Authors:** Nouf N. Mahmoud, Lubna M. Al-Kharabsheh, Enam A. Khalil, Rana Abu-Dahab

**Affiliations:** 1 Faculty of Pharmacy, Al-Zaytoonah University of Jordan, Amman 11733, Jordan; 2 School of Pharmacy, The University of Jordan, Amman 11942, Jordan

**Keywords:** human dermal fibroblasts, gold nanorods, cytotoxicity, cellular uptake, wound healing, interlukin-1β

## Abstract

Herein, the cytotoxicity, cellular uptake and wound healing of human dermal fibroblasts were investigated upon treatment with gold nanorods (GNR) decorated with different ligands. Neutral and cationic poly ethylene glycol (PEG)-decorated GNR demonstrated the least cytotoxicity and cellular internalization, while anionic- and bovine serum albumin (BSA)-coated GNR revealed significant cytotoxicity and cellular uptake into human dermal fibroblasts. The cell scratch test demonstrated that neutral, cationic PEGylated GNR and anionic-decorated GNR have accelerated the wound healing rate in vitro after 24 h of incubation with scratched human dermal fibroblasts compared to control, while there was a drastic retardation of wound healing rate of scratched fibroblasts upon exposure to BSA-GNR accompanied with a significant release of the inflammatory cytokine; interlukin-1β (IL-1β). The cytotoxicity of GNR against the dermal cells and their ability to enhance the wound healing in vitro are greatly linked to their surface modifications.

## 1. Introduction

Nanomedicine poses beneficial applications in drug delivery, imaging, diagnosis and treatment of several diseases [1,2,3]. Understanding the interaction of nanomaterials with skin upon intended or unintended exposure is crucial for evaluating their safety and potential biomedical applications [4]. Evaluating the uptake and deposition of nanoparticles into the different layers of skin is essential to assess the skin-nano interface [5]. 

Gold nanoparticles (GNP) particularly, gold nanorods (GNR) own several fascinating optical merits which make them attractive in nanomedicine and biomedical applications such as imaging, sensing, diagnosis and photothermal-based therapy [6,7,8,9,10]. Interaction of GNP of different shapes, sizes and surface modifications with skin was investigated in several published articles [8,11,12,13]. Probing the interaction of GNP with skin tissue at the cellular level is commonly performed by using human dermal fibroblasts, which are responsible for connective tissue production and for skin recovery [14] and they are implicated in the wound healing process [15]. Keratinocytes, the major cells in the epidermis, are also commonly used as a model in wound healing studies. Keratinocytes have a pivotal role in wound healing through their contribution in re-epithelization process [16], and it is the cross talk between fibroblasts and keratinocytes that initiate and maintain cell migration and wound healing [17].

Non-spherical GNP have various advantages over spherical counterparts in skin nanomedicine such as treatment and diagnosis of skin infections, wounds and skin cancer due to their unique photothermal and antibacterial activities [8,9,10,18,19,20].

Various studies have been conducted to evaluate the cytotoxicity of GNP of different surface modifications against human dermal fibroblasts, however, no conclusive results were provided. Furthermore, evaluating the cytotoxicity and cellular uptake of non-spherical GNP such as GNR upon exposure to human dermal fibroblasts was very limited in the literature. 

Pernodet et al., suggested a dramatic changes in cell activities of human dermal fibroblasts upon exposure to citrate-coated GNP as a result of their high cellular uptake [21]. Also, cytotoxicity in human dermal fibroblasts was mediated by oxidative stress and was independent of nanoparticle size upon treatment with spherical shape of GNP [22]. On the other hand, Coradeghini et al., have found that citrate-coated GNP of 5 nm, but not 15 nm, revealed cytotoxicity against mouse fibroblasts [23]. Besides, Bhamidipati et al., demonstrated that the surface modification of GNP has a detrimental role in their cytotoxicity against healthy and cancerous cells, despite their sizes or morphology, whereas CTAB-coated GNP and PEGylated nanostars demonstrated the highest and lowest cytotoxicity, respectively [24]. 

The effect of GNP on skin wound healing has been investigated in vitro using human dermal fibroblasts and keratinocytes as models, and their effect on the release of several inflammatory markers was evaluated too [25,26,27,28]. Recently, GNR loaded into poloxamer 407 hydrogel significantly accelerated the wound healing effect in vivo and dramatically reduced the gene expression of pro-inflammatory markers [19]. In another study, fibroblasts demonstrated reduced wound healing rate upon exposure to metal nanoparticles [29], while Pivodová et al. revealed that GNP were non-toxic to fibroblasts and keratinocytes and they reduced the production of pro-inflammatory cytokines such as IL-6, IL-12 and TNF-α, and other proteins involved in angiogenesis [30].

In this study, the role of surface chemistry of GNR on their cytotoxicity against human dermal fibroblasts was evaluated. In addition, their cellular uptake was investigated using inductively-coupled plasma-optical emission spectroscopy (ICP-OES) and transmission electron microscope (TEM) imaging. The wound healing ability of human dermal fibroblasts upon treatment with GNR of different surface modifications, and their effect on production of the inflammatory mediator, Interleukin-1β (IL-1β), were studied in this work.

## 2. Materials and Methods

### 2.1. Synthesis, Functionalization and Characterization of GNR of Different Surface Modifications

GNR were synthesized using a mixture of cetyltrimethylammonium bromide (CTAB) and oleic acid according to a previous protocol [31] with slight modifications [32]. The prepared GNR were modified using thiol containing ligands, a negatively charged polymer and a protein in order to obtain GNR with different surface chemistries. 

The following ligands (purchased from Sigma-Aldrich Chemicals, St. Louis, MO, USA) were utilized to modify the surface of GNR as described previously: poly acrylic acid (PAA, Mw ~15,000) [10], methoxy (m)-poly ethylene glycol thiol (m-PEG-SH, Mw ~2000) [32], amine (NH_2_-PEG-SH, Mw ~2000) [8] and bovine serum albumin (BSA). Milli-Q water was used to prepare the GNR suspensions.

GNR were functionalized with BSA by dispersing 10 mL of diluted GNR with 1.0 mL of BSA aqueous solution (10 mg/mL) [33]. The solution was kept under stirring at room temperature for 24 h followed by centrifugation twice. The obtained BSA-GNR pellets were re-suspended in milli-Q water and stored at −4 °C. 

The surface modified GNR were characterized in terms of UV-vis absorption spectroscopy using UV–vis spectrophotometer (UV-Vis Spectrophotometer, Shimadzu UV-1800, Kyoto, Japan) over the wavelength range from 400 to 1100 nm. Zeta potential and hydrodynamic size of the prepared GNR were measured using size/zeta analyzer (Nicomp Nano Z3000 particle size/zeta potential analyzer, Santa Barbara, CA, USA). Purified and diluted samples of GNR were transferred into dynamic light scattering (DLS) cuvettes for size measurement or folded capillary cells for zeta potential measurement. The cells were put into DLS at 25 °C and the hydrodynamic diameters of the samples and their surface effective charges were measured. The results are stated as mean ± standard deviation (SD) from three independent experiments. TEM imaging was performed using FEI Morgani 268, operating voltage of 60 kV, Eindhoven, The Netherlands. The diluted samples of GNR suspensions were dried over Formvar-coated copper TEM grids (300 mesh, Electron Microscopy Sciences, Hatfield, PA, USA). BSA-GNR sample was stained with uranyl acetate stain (Fluka Chemie, Buchs, Switzerland) for TEM imaging.

### 2.2. Human Dermal Fibroblasts Culture Conditions

Human dermal fibroblasts CCD-1064Sk (ATCC^®^ CRL-2076TM, Manassas, VA, USA) were cultured in Iscove’s Modified Dulbecco’s Medium (IMDM, Euroclone, Pero MI, Italy) supplemented with 10% *v*/*v* fetal bovine serum (FBS, Euroclone, Pero MI, Italy) and 1% *v*/*v* of 200 mM L-glutamine. Penicillin-Streptomycin; 100 IU/mL–100 µg/mL (Euroclone, Pero MI, Italy) was added to the media. Cells were kept at 37 °C in a humidified atmosphere containing 5% CO_2_ and used at a confluence of 80–90%.

### 2.3. Evaluation of the Cytotoxicity of GNR of Different Surface Modifications on Human Dermal Fibroblasts

#### Cellular Viability Assay

The cellular viability of human dermal fibroblasts was evaluated upon treatment with GNR using MTT (3-(4,5-dimethylthiazol-2-yl)-2,5-diphenyltetrazolium bromide) assay [34]. Human dermal fibroblasts were seeded at density of 2 × 10^4^ cells/well in a 96 well plate and incubated with 100 μL supplemented IMDM until the confluency reached 80–90%. GNR suspensions were serially diluted with IMDM without fetal bovine serum (FBS) to obtain different concentrations of GNR (0.0078, 0.01562, 0.03125, 0.0625 and 0.125 nM). The cells were incubated with the treatments for 72 h, after incubation, the supernatant was aspirated, then 100 μL of fresh IMDM was added and cells were incubated for 30 min at 37 °C. MTT solution (5 mg/mL, 10 μL, Sigma-Aldrich Chemicals, St. Louis, MO, USA) was added to the wells and incubated at 37 °C. After 3 h, supernatant was aspirated and 100 μL of dimethyl sulfoxide (DMSO, Euroclone, Pero MI, Italy) was added to solubilize the formed blue formazan crystals using shaker for 10 min at 120 rpm. The absorbance was recorded at 570 nm using microplate reader (Biotek Instruments, Winooski, VT, USA) and the cellular viability was calculated relative to the control.

### 2.4. Evaluation of the Cellular Uptake of GNR of Different Surface Modifications into Human Dermal Fibroblasts

#### 2.4.1. Quantitative Measurement of GNR Accumulated into Cells by ICP-OES

Human dermal fibroblasts were grown using IMDM supplemented with FBS in tissue culture flasks (25 cm^2^) till 80–90% confluency. On the experimentation day, GNR suspensions were diluted in IMDM without FBS (0.10 nM) and were placed onto the cells and the flasks were then incubated at 37 °C for 2 h. Fibroblasts incubated with only IMDM served as a control. The treated cells were taken out from the incubator after 2 h, and each flask was treated as following for quantitative analysis by ICP-OES: Trypsin-EDTA (1X, Biowest, Riverside, MO, USA) (1 mL) was added to each flask and the flasks were then incubated at 37 °C for 10 min. After that, 3.0 mL of IMDM were added to each flask to stop the enzyme activity. The media were aspirated and centrifuged (1400 rpm, 4 °C, 30 min) until the pellets were formed. To each of the resulting pellets, 0.5 mL of freshly prepared aqua regia (HNO_3_:HCL; 1:3) was added to enhance the samples digestion overnight. The samples were then diluted with milli-Q water up to 5 mL and were then filtered using 0.22 μm Teflon filters. The concentrations of the accumulated gold into cells were quantified by ICP-OES (Quantima, GBC Scientific Equipment, Braeside VIC, Australia) using a well-validated method. Gold ions were measured and quantified at a wavelength of 242.795 nm and the gold concentration was calculated against a calibration curve of gold standard (0.2–10.0 ppm) for ICP (1000 ppm, Sigma-Aldrich Chemicals, St. Louis, MO, USA). The percentage of gold accumulated into the cells relative to the initial quantity of gold was expressed as mean ± SD from at least three independent experiments. 

#### 2.4.2. TEM Imaging of Human Dermal Fibroblasts Treated with GNR Suspension

Cells were grown using IMDM supplemented with FBS in 96 well plate until 90% confluency. Cells were treated with PAA-GNR diluted with IMDM (0.1 nM) and incubated for 6 h at 37 °C. The media was then aspirated and 50 μL Trypsin-EDTA was added at each well and the plate was incubated for 10 min at 37 °C, then, 100 μL of IMDM was added to each. The cells were then centrifuged at 1400 rpm at 4 °C for 15 min. The resulting cell pellets were fixed using 0.5 mL solution containing glutaraldehyde 3% (Sigma-Aldrich Chemicals, St. Louis, MO, USA) in phosphate buffer (0.2 M, pH 7.4). Cells were then washed twice in PBS (0.2 M, pH 7.4) and post fixed for 2 h in an osmium tetraoxide buffer (2%). Next, cells were dehydrated in an ascending ethanol series and were cleared in propylene oxide, left in 1:1 epoxy: propylene oxide overnight then left in pure epoxy overnight. Cells were then embedded in epoxy resin, cross sectioned using ultra-microtome into 70-nm thin sections, placed onto Formvar copper grids and imaged by TEM.

### 2.5. Evaluation of Wound Healing of Human Dermal Fibroblasts upon Exposure to GNR of Different Surface Modifications

#### 2.5.1. Human Dermal Fibroblasts Scratch Assay

Human dermal fibroblasts were seeded at density of 30 × 10^4^ cells/well in a 6-well plate using 4 mL of supplemented IMDM, then the cells were incubated until confluency 80–90%. A scratch was induced into the cells using the tip of micropipette and the wells were treated with the following GNR suspensions: PAA-GNR (0.0312 nM), PEG-GNR (0.125 nM), PEG-NH_2_-GNR (0.125 nM) and BSA-GNR (0.078 nM). The concentrations that showed 80% survival rates in comparison to control on the cellular viability assay were used in this experiment. Scratched untreated cells were served as a control. Pictures of the treated scratched cells were taken using AxioCamICc 5 on primovert microscope (Zeiss, Oberkochen, Germany) at 0, 24 and 48 h post treatment. The pictures were compared with the control and with each other. Three independent experiments were performed.

#### 2.5.2. Human Dermal Fibroblasts Cytokine (IL-1β) Release

##### Samples Preparation for IL-1β Release Analysis and Detection of Human IL-1β Release

The cells were seeded at density of 50 × 10^4^ cells/well in 6-well plates using supplemented IMDM (4.0 mL) in each well and incubated until confluency 80–90%. The confluent cells were washed twice using 1.0 mL of PBS. The cells were covered with 800 μL of IMDM and were incubated with freshly prepared suspensions of PAA-GNR (0.0312 nM), PEG-GNR (0.125 nM), PEG-NH_2_-GNR (0.125 nM) and BSA-GNR (0.0156 nM). In order to encourage the release of human IL-1β, the cells were scratched using the tip of a micropipette. The plates were incubated for 30 min and 60 min at 37 °C, then the samples were collected separately in small Eppendorf tubes with 400 μL in each and were preserved at −4 °C ready for analysis. Three independent experiments were performed. 

Human IL-1β ELISA MAX^TM^ Deluxe kit (Invitrogen, Carlsbad, CA, USA) was used for measurement of IL-1β levels produced from the cells into the culture supernatants according to the manufacturer’s instructions. 

### 2.6. Statistical Analysis

Statistical analysis was performed using one-way analysis of variance (ANOVA) test followed by Tukey’s multiple comparisons test using GraphPad Prism, version 7.0.

## 3. Results and Discussion

### 3.1. Synthesis, Functionalization and Characterization of GNR of Different Surface Modifications

**Figure 1:**
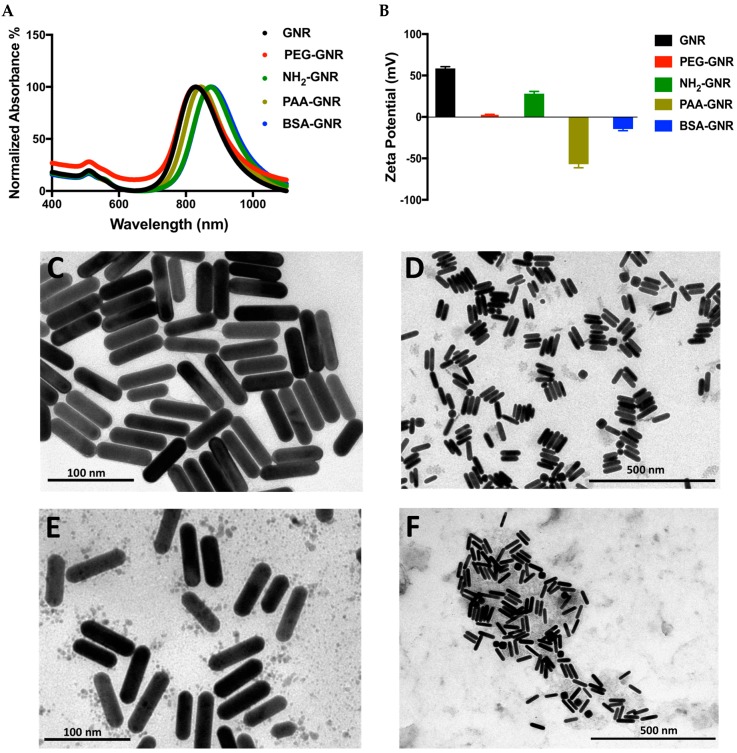
(**A**) UV-vis absorption spectra of GNR, PEG-GNR, NH_2_-GNR, PAA-GNR and BSA-GNR indicate typical transverse and longitudinal peaks of the nanorods. (**B**) Zeta potential of GNR of different surface modifications; GNR, PEG-GNR, NH_2_-GNR, PAA-GNR and BSA-GNR. (**C**) TEM image of PEG-GNR. (**D**) TEM image of NH_2_-GNR. (**E**) TEM image of PAA-GNR. (**F**) TEM image of BSA-GNR.

GNR suspension was synthesized following a previous protocol. A Colloidally stable GNR suspension was obtained with a typical optical spectrum of transverse and longitudinal peaks (Figure 1A). Upon surface modification of GNR with different chemical ligands, slight shift in their UV-vis spectra was observed without any significant tailing or broadening of their optical peaks suggesting their successful surface functionalization. The hydrodynamic size of GNR after surface functionalization was slightly increased, however, it was significantly increased after surface functionalization with BSA suggesting successful functionalization with the protein. The average zeta potentials of GNR, PEG-GNR, NH_2_-PEG-GNR, PAA-GNR, and BSA-GNR were +58.5, +2.3, +28, –57.0 and −14.5, respectively (Figure 1B). Furthermore, the morphology and size of GNR of different surface modifications were verified by TEM imaging. Figure 1C–E demonstrate TEM images of GNR decorated with PEG, PAA and NH_2_-PEG ligands, respectively and indicating the rod-shape of the nanoparticles with average length and width of 63.1 ± 3.1 nm and 15.0 ± 2.6 nm, respectively and an average aspect ratio (AR) of 4.2. TEM image of BSA-GNR indicates individuals or clusters of protein-coated nanorods (Figure 1F).

### 3.2. Cytotoxicity and Cellular Uptake of GNR of Different Surface Modifications against Human Dermal Fibroblasts

**Figure 2:**
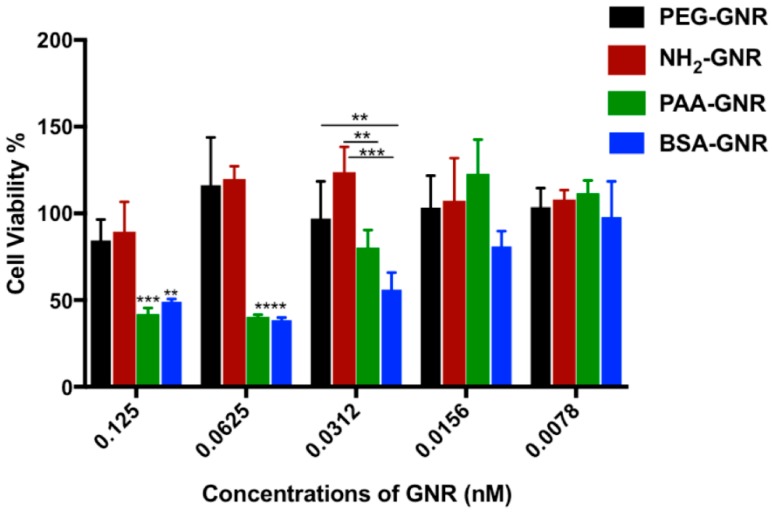
Cellular viability percentages of GNR of different surface modifications against human dermal fibroblasts. Anionic charged- and BSA-conjugated GNR demonstrated the highest cytotoxicity against human dermal fibroblasts. Data are presented as mean ± SD. One-way ANOVA was used to estimate the statistical differences; ** *p* < 0.01, *** *p* < 0.001 and **** *p* < 0.0001.

Cell culture is a commonly used model to assess the cytotoxicity of nanoparticles since molecular mechanisms of toxicity can be evaluated easily, however, the nanoparticles’ distribution into the body’s organs and tissues could not be taken into consideration. In this study, the cytotoxicity of GNR with diverse surface modifications towards human dermal fibroblasts was evaluated by estimating their cellular viability percentages. As shown in Figure 2, the prepared GNR suspensions demonstrated a concentration dependent cytotoxicity over the tested concentration range (from 0.0078 to 0.125 nM). The concentrations of GNR were selected based on their colloidal stability upon mixing with the tissue culture medium as confirmed by their colloidal color and optical spectra (not published observations). Generally, the cells incubated with low concentrations of GNR revealed high cellular viability (>80%). Interestingly, BSA-GNR and PAA-GNR demonstrated the highest cytotoxicity over the concentration range of 0.0312–0.125 nM, while neutral and cationic PEGylated nanorods revealed the highest cellular survival percentages for all the concentrations under investigation. Similarly, Bhamidipati et al. have found that PEGylated gold nanostars showed low cytotoxicity against healthy and cancerous cells [24]. 

**Figure 3:**
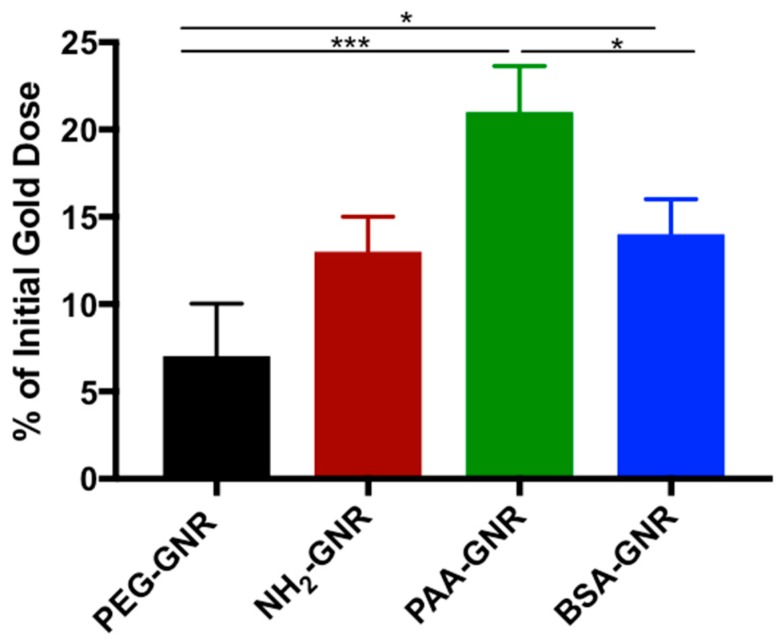
Percentages of GNR of different surface modifications accumulated into human dermal fibroblasts after 2 h of incubation. Data are presented as mean ± SD. One-way ANOVA was used to estimate the statistical differences; * *p* < 0.05, ** *p* < 0.01 and *** *p* < 0.001.

The above results of cytotoxicity are well correlated with the cellular uptake findings (Figure 3), where the percentages of GNR accumulated into the fibroblasts relative to the initial GNR quantity were estimated. Neutral PEGylated GNR showed the least cellular accumulation percentage (~7.0%) into human dermal fibroblasts, while negative charged PAA-GNR demonstrated the highest cellular uptake percentage into the cells (~21%), which is most likely responsible for their observed high cytotoxicity against human dermal fibroblasts. In line with our results, Grabinski et al. have demonstrated that PEG-GNR have minimal cellular viability activity towards HaCaT cells and demonstrated low cellular uptake [35]. Cationic PEGylated GNR (NH_2_-GNR) and BSA-GNR demonstrated similar percentages of cellular uptake (12% and ~14%, respectively), however, their cytotoxicity was not similar. 

Alkilany et al., have found that anionic and cationic GNR exhibited similar anionic surface charge upon mixing with the tissue culture media due to formation of protein corona and they demonstrated similar cytotoxicity against the aortic endothelial cells [36]. Furthermore, they found that PEGylation of GNR enhanced the biocompatibility of the nanoparticles. 

On the other hand, cationic nanoparticles exhibited high cytotoxicity compared to anionic or neutral charged nanoparticles due to their enhanced penetration through the cell membrane as described in several reports and studies in the literature [37,38,39,40]. 

These conflicting results concerning the cytotoxicity of GNR with different surface modifications may originate from using different cell lines (cancerous vs. normal cells), type and composition of the tissue culture media and the colloidal stability of the nanoparticles upon exposing to the tissue culture media and whether proteins were added to the media during cell viability testing or not. The presence of proteins in the tissue culture media greatly contributes to protein corona formation and may affecting the surface charge and size of the nanoparticles and ultimately enhancing or retarding their cellular uptake and cytotoxicity [34]. Accordingly, we believe that our current work provides systematic insight into the interaction of GNR of different surface functionalities with fibroblasts in terms of cellular intake, cytotoxicity and ability to heal wounds in vitro. Importantly, these studies were performed under the same parameters of cell type, tissue culture medium and its composition, incubation time and concentration of GNR to eliminate any possible artifacts of the findings.

In parallel, we measured the zeta potential of GNR upon mixing with tissue culture medium containing no protein for 24 h, and we found that minimal change in zeta potential values was observed for all used GNR suspensions (results are not shown).

The cellular uptake mechanism of GNP is highly dependent on their sizes, shapes and surface modifications, and different mechanisms of cellular internalization were discussed in the literature. For example, transferrin-coated GNR were up taken via receptor-mediated endocytosis (RME) pathway [41], while the cellular uptake mechanisms of small cationic GNP involved caveolae and dynamin pathways [42]. Jiang et al., demonstrated that the cellular uptake of cationic and anionic nanoparticles was mediated by endocytic pathways [43]. Ding et al., found that the cellular internalization of 15 nm and 45 nm GNP was mediated via RME pathway, while the 80 nm GNP were internalized into the cells by micropinocytosis. Furthermore, they found that formation of protein corona can greatly alter the cellular uptake mechanism of GNP [44]. In addition, the shape of nanoparticles greatly affects their cellular uptake. For example, Xie et al. have found that gold nanotriangles demonstrated the highest cellular uptake into RAW264.7 cells, followed by GNR and gold nanostars. They revealed also that the cellular uptake of GNP was induced by different endocytosis mechanisms, dependent on the shape of nanoparticles [45]. 

In our study, coating the nanorods with BSA enhanced their cellular uptake into the fibroblasts and consequently their cytotoxicity. Similarly, Holt et al., indicated that BSA-stabilized single-wall carbon nanotubes were dramatically accumulated into human mesenchymal stem cells and HeLa cells [46]. Besides, the cellular uptake of albumin-coated nanodiamond into lung cancer cells was mediated by selective autophagy as described by Lawrence et al. [47]. Importantly, Li et al., found that BSA-GNP had been transported into lysosomes and resulted in G2/M arrest by microtubule stabilization similar to many anti-cancer drugs. Such findings support our results regarding the dramatic cytotoxicity of BSA-GNR [48].

The cellular viability of the cells at some points was more than 100%, which is highly common in the literature due to many reasons such as interference with mitochondrial dehydrogenase activity [49].

### 3.3. TEM Imaging of Human Dermal Fibroblasts Treated with GNR Suspension

**Figure 4:**
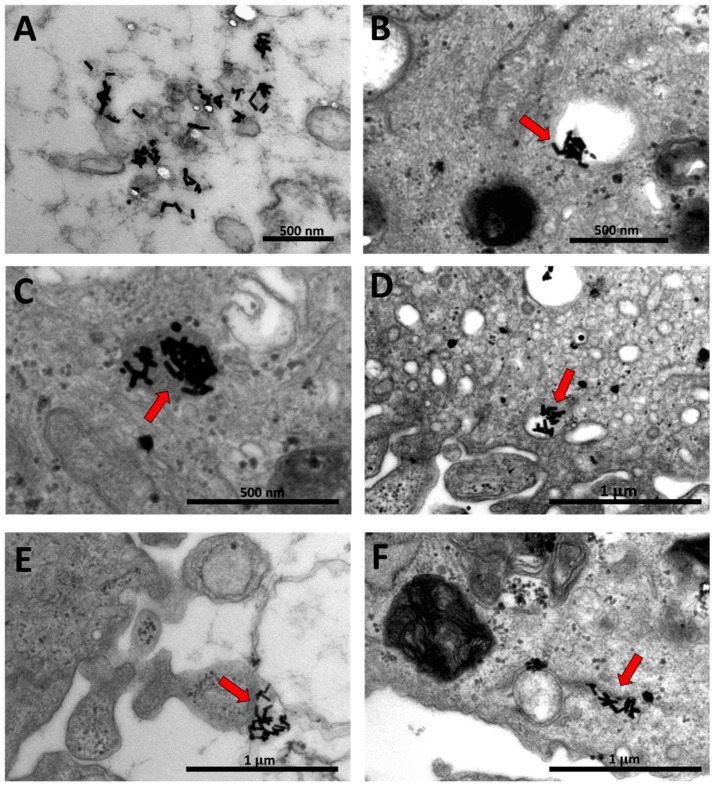
TEM images of human dermal fibroblasts upon treatment with PAA-GNR. Individuals and clusters of nanorods were accumulated into different regions of the cells; into the cytoplasm (**A**,**C**,**F**), into the vacuoles (**B**,**D**) and at the cell membrane (**E**).

In order to confirm the cellular uptake of GNR, TEM imaging was performed for the anionic charged GNR as they revealed the highest percentage of cellular accumulation into the fibroblasts and the highest cytotoxicity. Figure 4A–F showed the human dermal fibroblasts containing a large number of penetrated nanorods accumulated into different regions of the cells as individuals or clusters and maintained their shape. Favi et al., indicated that GNR in general are up taken by membrane bound vesicle method [50] and anionic nanoparticles particularly are up taken through the interaction with the positive site of the proteins in membrane, and they can be highly captured by cells because of their repulsive interactions with the negatively charged cell surface [51]. 

### 3.4. Wound Healing Ability of GNR of Different Surface Modifications

**Figure 5:**
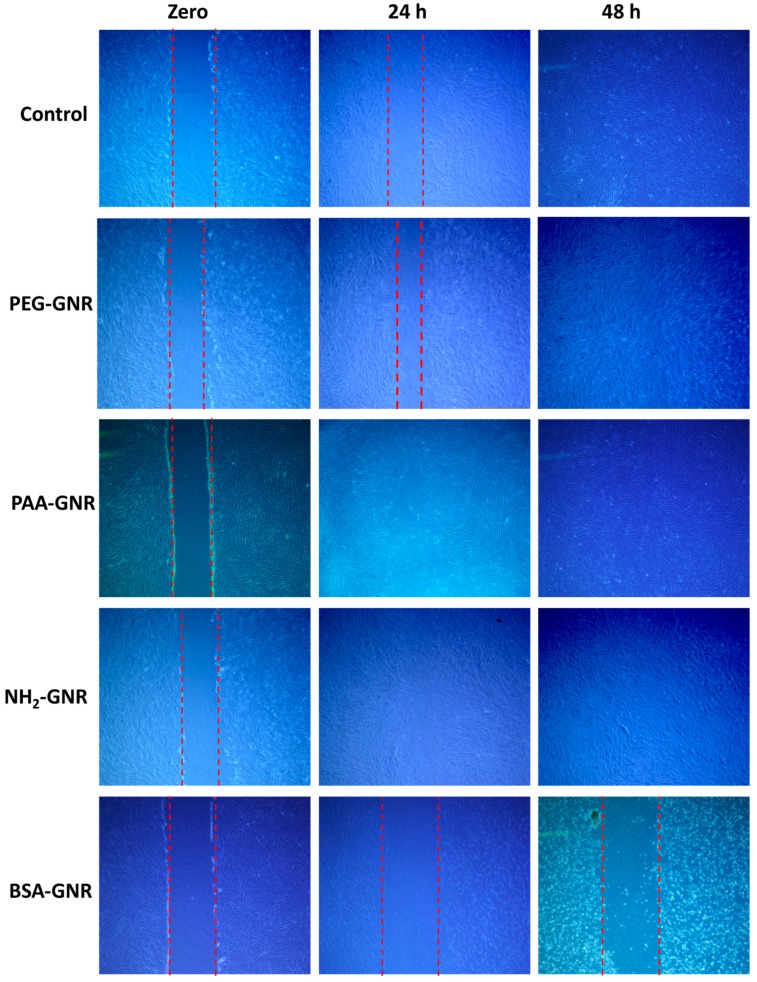
Wound healing ability of GNR of different surface modifications towards human dermal fibroblasts at different times of incubations. PEG-GNR, PAA-GNR and NH_2_-GNR enhanced the wound healing of scratched fibroblasts after 24 h of incubation compared to control, while BSA-GNR significantly retarded the wound healing of fibroblasts after 24 h and 48 h of incubation.

Healing of the wounds is a sophisticated process mediated by a system of signaling pathways that synchronize many cellular processes, such as migration and proliferation, which will lead to wound closure. Wound healing is a firmly controlled process, any changes in any constituent of this process can be unfavorable leading to additional tissue damage or poor healing [52]. Keratinocytes and fibroblasts are the most common cell models used for wound healing studies in vitro. In this study, the effect of GNR of different surface functionalities on wound healing was investigated in vitro. A scratch assay was performed on the monolayers of cells that were incubated with or without GNR suspensions. After introducing a “scratch” or “wound” into a cell culture, images were captured immediately, 24 h and 48 h after the scratch. Figure 5 indicates that upon cell scratch; cells migrate trying to enhance wound closure by elongating themselves. Clearly, PEG-GNR showed slightly faster wound healing rate than control after 24 h of incubation (Average percentage of wound size reduction; ~25% vs. ~14%, respectively), while PAA-GNR and NH_2_-PEH-GNR demonstrated accelerated wound healing rate after 24 h of incubation (Average percentage of wound size reduction; ~32% and ~50%, respectively) compared to control. Although anionic GNR showed high cytotoxicity, they accelerated healing of the scratched fibroblasts. This is might be because the concentrations used in this experiment showed 80% survival rates in comparison to control. On the other hand, BSA-GNR demonstrated obvious cytotoxicity and retarded the wound healing of the scratched fibroblasts compared to control (Average percentage of wound size reduction; ~0.1% vs. ~14%, respectively). These results are consistent with the previous cellular viability assay findings, where BSA-GNR demonstrated high cytotoxicity against human dermal fibroblasts. In agreement with these results, our recent study demonstrated a dramatic wound healing of PEG-GNR and cationic poly allyl amine hydrochloride-coated GNR loaded into poloxamer 407 hydrogel in rats. Also, we found that such nanorods have accelerated the gene expression of anti-inflammatory markers and exhibited potent antibacterial activity against the most common skin pathogens [19]. 

The release of IL-1β as an inflammatory marker upon scratching the cells was investigated upon treatment with GNR of different surface modifications. IL-1β is a cytokine induced by damaged cells and responsible for inflammation and fibrosis [53].

**Figure 6:**
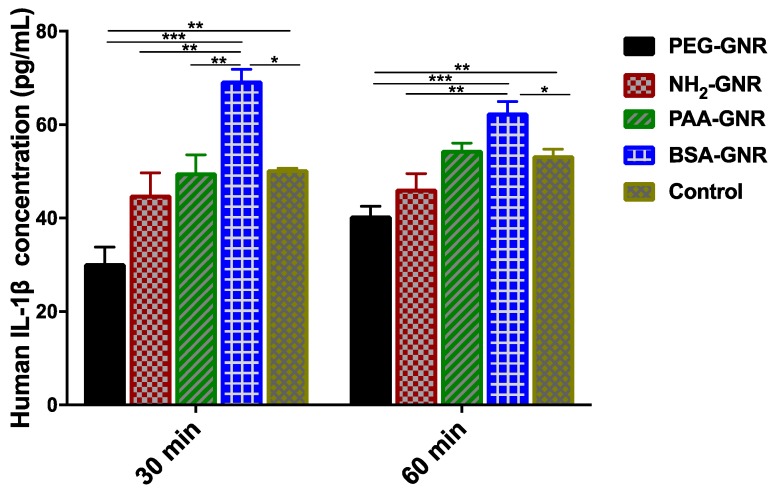
Concentrations of IL-1β inflammatory cytokine released upon exposure of scratched human dermal fibroblasts to GNR of different surface modifications at different time points. PEG-GNR significantly reduced the production of IL-1β inflammatory cytokine while BSA-GNR significantly enhanced its production upon exposure to scratched human dermal fibroblasts. Data are presented as mean ± SD. One-way ANOVA was used to estimate the statistical differences; * *p* < 0.05, ** *p* < 0.01 and *** *p* < 0.001.

Results of IL-1β production demonstrated that the release of IL-1β was significantly increased in human dermal fibroblasts treated with BSA-GNR compared to control and other treatments after 30 min and 60 min of cell scratch (Figure 6). On the other hand, the release of IL-1β was significantly reduced in scratched fibroblasts treated with PEG-GNR, while its release was not significantly increased compared to control in scratched fibroblasts treated with anionic or cationic GNR (Figure 6). These results are well correlated with the previous observed obvious cytotoxicity of BSA-GNR against human dermal fibroblasts. Although cationic and anionic charged GNR revealed no significant effects on the release of IL-1β marker, their positive impact on wound healing could not be excluded and could be mediated by other pathways. 

Similarly, Dong et al. suggested that BSA-coated GNR induced reactive oxygen species (ROS)-dependent apoptosis in HepG-2 cells [54]. Moreover, Grabinski et al. found that mercaptohexadecanoic acid-coated GNR induced significant upregulation of genes associated with inflammation in HaCaT cells, which increased the production of cytokines such as IL-1 family, while exposure to PEG-GNR encouraged down-regulation of genes linked with apoptosis, DNA damage, and growth arrest [35]. 

## 4. Conclusions

Understanding the dermal-nano interface is crucial to estimate the toxicity and therapeutic effects of nanoparticles upon topical exposure. Although cytotoxicity of surface-functionalized gold nanoparticles such as PEG- and BSA-coated GNP and other surface chemistries was reported in the literature, the results are highly conflicting. In this study, a systematic insight into the interaction of GNR of different surface modifications with fibroblasts in terms of cytotoxicity, cellular uptake and wound healing in vitro was provided. In summary, the cellular viability and uptake of GNR into human dermal fibroblasts were greatly dependent on their surface decorations. PEGylated nanorods demonstrated the least cytotoxicity and cellular uptake compared to anionic or BSA-coated GNR which demonstrated significant cytotoxicity and cellular internalization. PEGylated and charged-GNR accelerated wound healing rate of scratched human dermal fibroblasts in vitro, while BSA-coated counterpart caused drastic cytotoxicity and dramatically retarded the wound healing of the scratched fibroblasts accompanied with significant release of IL-1β inflammatory cytokine.
