# Correction: Mahmoud et al. Interaction of Gold Nanorods with Human Dermal Fibroblasts: Cytotoxicity, Cellular Uptake, and Wound Healing. *Nanomaterials* 2019, *9*, 1131

**DOI:** 10.3390/nano11061364

**Published:** 2021-05-21

**Authors:** Nouf N. Mahmoud, Lubna M. Al-Kharabsheh, Enam A. Khalil, Rana Abu-Dahab

**Affiliations:** 1 Faculty of Pharmacy, Al-Zaytoonah University of Jordan, Amman 11733, Jordan; 2 School of Pharmacy, The University of Jordan, Amman 11942, Jordan; lubna.mohammadkh@gmail.com (L.M.A.-K.); abudahab@ju.edu.jo (R.A.-D.)

The authors wish to make the following correction to Figure 1 D in this paper [1]. Replace Figure 1 with:


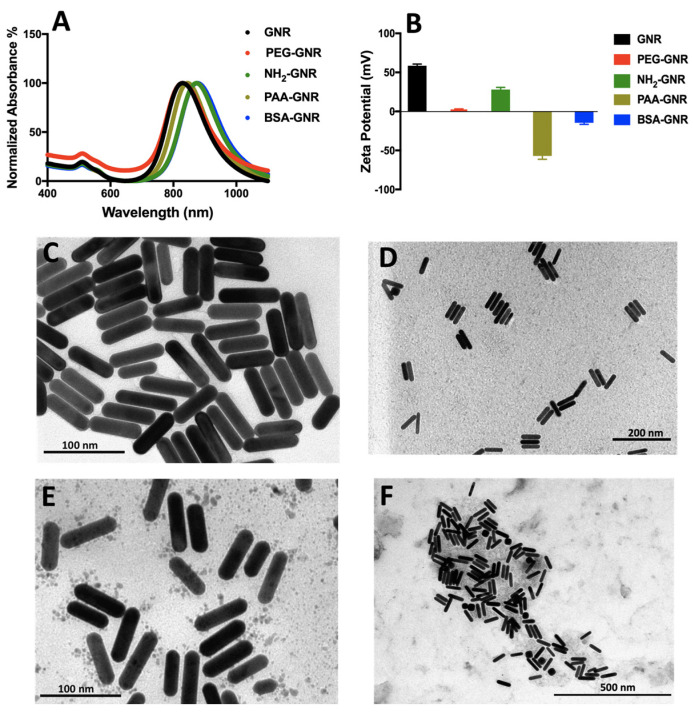


The authors would like to apologize for any inconvenience caused to the readers by this change.
